# The climate emergency: A tale of two cities

**DOI:** 10.1016/j.heliyon.2024.e24294

**Published:** 2024-01-20

**Authors:** Judy Too, Obuks A. Ejohwomu, Felix K.P. Hui, Nilupa Herath, Colin Duffield

**Affiliations:** a Department of Mechanical, Aerospace and Civil Engineering, University of Manchester, Engineering Building A, Booth Street E, Manchester, M13 9PL, United Kingdom; b Department of Infrastructure Engineering, University of Melbourne, Melbourne Connect, Carlton, VIC, 3053, Australia

**Keywords:** Building, Climate change, Climate emergency, Performance-based culture, Zero carbon

## Abstract

The rising concentration of greenhouse gases (GHGs) and the associated impacts of climate change have prompted the urgent need to reduce GHG emissions. In response, the cities of Manchester and Melbourne declared climate emergencies in 2019, calling for radical resource mobilisation to address global warming. Despite the increasing discourse on climate change policies and ambitions, there is limited evidence on the current practices in the built environment following these climate emergency declarations. To address this gap, this research conducted a comparative analysis of the building sector practices in Manchester and Melbourne. Through 63 in-depth interviews with building professionals and policy experts, this study sheds light on the alignment of these practices with climate goals. The findings reveal that while the United Kingdom has made notable progress in establishing its net-zero pathway, little attention has been given to driving this transformation at the building project level. In Melbourne, stricter regulations are necessary, particularly in the residential sector, to facilitate emission reductions and behavioural change. This paper, therefore, proposes a holistic cultural reform framework to support the transition of the sector towards a performance-based culture. By contextualising this analysis within the broader policy landscape and gathering insights from building professionals and policy experts, this research contributes to global efforts in climate change mitigation and offers practical implications for the building sector.

## Highlights

• Periodic reviews are necessary to evaluate the impact of decarbonisation decisions.
• Building sector should adopt a performance-based culture to meet climate targets.
• A holistic cultural reform framework is proposed for a performance-based culture.

## 1 Introduction

Greenhouse gas (GHG) concentrations have increased substantially since the start of the industrial revolution, rising from an average annual concentration of 280 parts per million (ppm) in the late 1700s to 414 ppm in 2021 [1]. This increased levels of GHG trap heat in the atmosphere, raising the earth's average temperature by approximately 1.1° Celsius compared to the pre-industrial era [2]. Consequently, different regions across the globe are currently experiencing frequent extreme weather events, with devastating impacts on individuals and communities [3].

Cities are pivotal players in this climate crisis, generating significant GHGs from the combustion of fossil fuels for heating and cooling buildings, construction, transport and industrial processes [4]. Despite their contribution to the crisis, cities are also recognised as the intersection point of various sectors, offering substantial potential for synergistic climate-neutral strategies [5]. In line with the Intergovernmental Panel on Climate Change' s (IPCC) recommendations to achieve net-zero emissions by 2050 to limit global warming to 1.5° Celsius above pre-industrial levels [6], over 2327 jurisdictions in 40 countries have acknowledged the urgent need for climate action and declared climate emergencies [7]. Notable among these are Manchester and Melbourne, both of which declared climate emergencies in 2019, calling for immediate and radical mobilisation of resources to combat global warming [8,9].

The building sector is particularly crucial because, on one hand, it is one of the largest contributors to GHGs, and on the other hand, it is exposed to the risks of climate change [10]. The interplay between the built environment and urban development exerts a significant influence on the global climate change phenomenon and its consequential adverse environmental effects. Notably, the built environment is accountable for approximately 40 % of global GHGs [11]. Despite evolving scholarly discourse on climate change policies and ambitions at the national and city levels (e.g. Refs. [12,13]), there is a notable absence of comprehensive evidence on the current building sector practices in response to climate emergency declarations. It is also worth noting that the majority of studies in this research area tend to focus on either the supply-side factors contributing to emission reduction (e.g. Ref. [14]), or the demand-side factors (e.g., Ref. [15]). To offer a stronger reference for global, national, and regional carbon reduction policies and actions, Yao et al. [16] recommends that research must provide holistic assessments of both supply-side and demand-side factors. This study addresses this research gap and positions itself as an essential contribution to the ongoing discourse on the built environment response to the climate emergency. Specifically, it examines the building sector practices in Manchester (United Kingdom) and Melbourne (Australia) to assess their alignment with the 2050 target. By comparing these practices, this research aims to answer the question: How do the building sector practices and underlying construction industry cultures in Manchester and Melbourne compare in their efforts towards achieving climate targets? In juxtaposing these two cities, this research provides the first opportunity for cross-country comparison of building sector practices, unravelling the multifaceted nature of carbon reduction measures and the *“push and pull”* forces in the supply chain.

This research effort contributes to two lines of inquiry. First, through in-depth analysis of the literature and subsequent validation via empirical data, this study responds to Millot et al.'s [17] recommendations for conducting cross-country comparisons that illuminate best practices, barriers and opportunities towards achieving climate goals. Given the variations in building sector culture and practices across different countries, a compelling inquiry is whether these disparities can be remedied through government policies and regulations on climate change. This paper therefore conducts a comparative analysis of two cities facing distinct climatic and political contexts. Notably, both the UK and Australia have legislated their long-term carbon reduction targets. However, the UK's legislation goes further by outlining specific policies and mechanisms to achieve those targets [18,19]. By exploring building sector culture and practices in these cities, this research extracts valuable lessons on the strategies employed to address the climate emergency and lays the groundwork for more effective cross-border knowledge sharing.

Secondly, this study offers a unique perspective by anchoring itself in the perspective of building sector experts, thereby complementing discussions among stakeholders, policymakers, and researchers on how to address the 2050 agenda in the building sector. Presently, it remains unclear whether the climate emergency plans developed by local governments reflect conventional responses or have culminated in more multifaceted solutions aimed at addressing this complex challenge [20]. This study, therefore, brings to the fore critical insights into the building sector practices vis-à-vis climate targets and proposes a holistic cultural reform framework as a way forward for transitioning from business-*as*-usual practices to performance-based practices. Apart from the research community, the findings have relevance for evidence-based policies on decarbonisation globally.

The rest of the paper is structured as follows: *Section two* provides a comprehensive literature review of building sector emissions highlighting the UK and Australia's climate change targets. *Section three* delves into the research methodology. *Section four* offers a comparative analysis of the two cities, highlighting the similarities, disparities, and influencing factors. Finally, *Section five* presents a synthesis of the key findings and the implications for policymakers, researchers and building sector practitioners, underscoring the need to adopt a performance-based culture in the construction industry.

## 2 Literature review

### 2.1 Building sector emissions

Buildings and construction are responsible for 37 % of global energy-related emissions [21]. Of these emissions, 27 % are attributed to direct emissions (Scope 1) and indirect emissions (Scope 2), while the remaining 10 % is associated with the production of materials and the construction of buildings (Scope 3). Building life cycle emissions have two main aspects: Operational and embodied emissions [22]. Operational emissions refer to the total direct (Scope 1) and/or indirect (Scope 2) emissions from the energy consumed during the use phase of the building lifecycle [23]. This includes both regulated loads such as heating, cooling, lighting, and ventilation as well as unregulated loads such as ICT equipment and refrigeration appliances [24].

Embodied emissions encompass the total direct and indirect emissions arising from the manufacturing of materials and construction processes [25]. These embodied emissions can be categorised into three distinct parts: initial, recurring and end-of-life emissions. The initial embodied emissions represent the initial energy expended in sourcing raw materials, processing, manufacturing and transportation to the construction site [26]. The energy expended in replacement processes during repair and renovation is referred to as recurring embodied energy, while the energy utilised in disposal processes represents the end-of-life embodied energy [27].

While building energy codes have matured over time, their primary focus has traditionally been on reducing operational energy [23]. Consequently, there has been limited adoption of mandatory codes governing embodied carbon performance [28]. The Netherlands is a notable exception, where regulations mandate the consideration of embodied carbon for all residential and commercial structures exceeding 100 m^2^ [29]. Similarly, France's RE2020 mandates maximum allowable embodied carbon values for new constructions, with residential units targeted to achieve less than 640 kgCO_2_e/m^2^ by 2024 and further decreasing to no more than 415 kgCO_2_e/m^2^ by 2031 and beyond [30]. In Australia, discussions are emerging to incorporate embodied emissions within the National Australian Built Environment Rating System (NABERS) framework [31], while in the UK, the proposal for Building Regulations ‘Part Z’ seeks to enforce the reporting and control of entire lifecycle carbon emissions [32]. Several organisations, such as the UK Green Building Council and the London Energy Transformation Initiative [33], have also responded by producing practical guidance on embodied carbon. Additionally, the Royal Institute of Chartered Surveyors (RICS) has released a Professional Statement outlining the methodology for assessing embodied and whole-life carbon in the UK, which aligns with EN 15978 and has been accepted as the industry methodology for whole lifecycle carbon assessments.

### 2.2 Emission reduction strategies in UK and Australia

#### 2.2.1 United Kingdom

The UK established itself as a leader in climate policy by enacting a net zero carbon emissions target in June 2019 [34]. To support this target, the Climate Change Act requires mandatory carbon reporting for organisations and underscores the country's commitment to reducing greenhouse gas (GHG) emissions by at least 80 % below 1990 levels by 2050 [35]. Achieving this ambitious goal requires the country to accelerate its decarbonisation efforts over the next three decades compared to previous years [36]. In response to this challenge, the use of coal has experienced a steep decline, with plans for its complete phase-out by 2025 [37]. Additionally, the country has implemented various strategies to further advance its decarbonisation efforts such as investments in carbon capture utilisation and storage, installing electric heat pumps in homes and phasing out petrol and diesel vehicles [38].

In the building sector, the UK has committed to a net-zero carbon emissions target by 2050, aligning with its broader aim of achieving net zero emissions across the entire economy [38]. This is intended to be achieved by improving the energy efficiency of buildings and implementing cost-effective heating solutions such as heat pumps and hydrogen boilers [39]. In response, The United Kingdom Green Building Council (UKGBC) has introduced a Net Zero Whole Life Carbon Roadmap. This roadmap defines net zero whole life carbon buildings as structures with both operational and embodied impacts that are either net-zero or negative over their designated service life [40]. This is achieved by first reducing operational and embodied emissions to the greatest extent possible, followed by offsetting any unavoidable emissions.

To facilitate progress towards these targets, the Royal Institute of British Architects (RIBA) [41] has set performance targets for the UK's built environment (refer to Table 1). Local governments, private sector investors, asset owners and developers are now responding to this increased pressure to decarbonise their assets while organisations are adopting responsible business models for post-pandemic recovery. For instance, The London Plan [42] has integrated energy efficiency requirements for new developments and major renovations and places a strong emphasis on achieving net zero carbon by reducing on-site emissions by at least 35% beyond building regulations.

**Table 1:** Royal Institute of British Architects (RIBA) 2030 Climate Change target metrics.

Targets	New build offices			New build schools			Domestic/Residential		
	**BAU**	**2025**	**2030**	**BAU**	**2025**	**2030**	**BAU**	**2025**	**2030**
**Operational energy (kWh/m**^**2**^**/y)**	130	<75	<55	130	<70	<60	120	<60	<35
**Embodied carbon (kgCO**_**2**_**e/m**^**2**^**)**	1400	<970	<750	1000	<675	<540	1200	<800	<625

Where BAU refers to business-*as*-usual.

#### 2.2.2 Australia

Despite constituting just 0.33 % of the global population, Australia ranks among the highest per capita GHG emitters worldwide [43]. Australia's contribution to global emissions is approximately 1.3 % [44], primarily due to its heavy reliance on coal [45]. Unfortunately, the nation's political landscape has consistently fallen short in implementing adequate measures to reduce emissions and foster a transition towards a low-carbon economy [46]. For instance, Australia's Nationally Determined Contributions (NDCs) under the Paris Agreement have been criticised as inadequate and not in alignment with the target of limiting global warming to less than 2 °C [47]. This reluctance to address the urgent need for climate action stands in stark contrast to the policies and strategies pursued by other countries such as Netherlands, France and Denmark [48]. While widespread consensus exists on the need to transition to decarbonised economies in countries like the UK, Australia's political landscape has been marked by controversy and persistent denial regarding climate change [49]. As a result, climate advocacy and activism have called for improved policy measures, increased investment in renewable energy, and divestment from coal [49].

Similar to the UK, Australia aims to achieve a carbon-neutral built environment by 2050 [50]. This will be realised through adaptations to the National Construction Code (NCC), which governs building energy efficiency standards. Research indicates that even modest improvements to the NCC, such as better sealing, double-glazed windows, improved insulation, external shading, more efficient equipment, and the use of electric heat pumps could yield substantial energy savings ranging from 19 % to 56 % [51]. Besides the NCC, GBCA has outlined a decarbonisation roadmap with ambitious objectives of achieving net-zero carbon in operations for new buildings by 2030 and existing buildings by 2050 or earlier [50]. This roadmap entails a 20 % reduction in embodied emissions by 2035, and a commitment to offset embodied emissions from new buildings by 2050. To achieve these ambitious targets, the built environment must confront several challenges stemming from a shortage of innovative strategies, insufficient communication and information exchange among stakeholders, and a slow approach to addressing sustainability [52].

## 3 Materials and methods

### 3.1 Research design and sampling logic

The extant literature falls short of comprehensively elucidating the building sector practices employed at the project level to achieve climate targets. To address this gap, an abductive research strategy was employed. This strategy recognises the iterative and cumulative nature of research and combines theoretical concepts with empirical findings [53]. Given the limited knowledge of the concepts under investigation, a qualitative inquiry was considered appropriate for in-depth exploration [54]. A cross-country comparative analysis was particularly chosen because it offers a rich understanding of the opportunities and challenges experienced across different nations, yielding valuable insights into this research area [18]. The considerable size and scope of the UK and Australian building construction industries designated these nations as highly desirable subjects for this study's analysis. Specifically, the cities of Manchester and Melbourne were selected because of their economic significance, significant urban development and investments in sustainable building practices. These major cities were taken to be representative of the building sector practices in their respective countries.

To ensure comprehensive and diverse insights into the impact of the current industry practices on the progress towards achieving climate targets, a series of interviews were conducted with participants from various professional backgrounds (refer to Table 2). This approach was chosen to address concerns regarding the generalisation, relevance, and replicability of findings [55]. An initial stakeholder mapping exercise was carried out to identify experts with knowledge and experience in building sector practices in the UK and Australia, with a specific focus on building projects in Manchester and Melbourne. This research employed a purposive sampling approach based on two criteria. First, the participants were required to have at least six years of experience in the building sector to ensure a comprehensive examination of practices both before and after the declaration of the climate emergency. Second, participants were chosen based on their involvement in delivering major building construction projects in Manchester or Melbourne to communicate and demonstrate industry practices in their respective cities.

**Table 2:** Demographic profile of experts.

Professional background	Number of participants		Years of experience
	Manchester	Melbourne	
**Sustainability experts**^a^	5	9	10–30
**Engineers**^b^	5	6	14–47
**Architects**	4	3	18–30
**Quantity surveyors/cost consultants**	5	2	6–20
**Policy experts**	3	4	10–26
**Project Management Professionals**	3	5	11–28
**Builders/Contractors**	3	2	16–21
**Facility Managers**	2	2	19–25

a Sustainability experts included energy assessors, carbon consultants, climate change experts and sustainability leads.

b Engineers covered civil, structural, mechanical, and building services engineers.

### 3.2 Data collection

This research effort employed both primary and secondary data sources to investigate building sector practices and their implications towards achieving climate targets. Primary data was collected through semi-structured interviews. Semi-structured interviews were chosen because they enabled the participants to discuss their interpretations of the world in which they live and to express how they regard situations from their own point of view [56]. In academic research, the semi-structured interview holds distinct advantages. Firstly, it allows researchers to gather in-depth information and evidence from interviewees while staying aligned with the study's focus [57]. Secondly, it offers flexibility and adaptability compared to unstructured interviews, where the interview direction is less controlled [58]. Recognising the complex interplay between policies, decision-making and stakeholders' perspectives [59], the semi-structured interviews were aimed at understanding the participants' practices in delivering new build projects. The in-depth probing was done to gather specific information from the participants and provide insights into the participants' experiences. To facilitate this, an interview protocol was employed, comprising a predefined set of questions to guide one-on-one interactions with the participants, following the recommendations of Larkin et al. [60]. The questions focused on the current building sector practices and the systems in place to comply with national climate targets. The participants offered insights into the supply chain intricacies, opportunities and challenges faced by the building sector towards achieving climate targets. Before commencing data collection, ethics clearance was obtained.^1^ Prior to each interview session, all study participants provided explicit informed consent. The interviews, which lasted between 1.5 and 2 h, were conducted in an open, consistent, and flexible manner using a pre-designed interview protocol, with care taken to avoid omitting key topics relevant to the study. All responses were then recorded and kept confidential. The interviews were conducted until a point of data saturation was reached, where new information produced little or no change to the codes and emerging themes. Data saturation, as recommended by Guest et al. [61]; signifies the stage at which the accumulation of new data ceases to yield new themes or insights. By adhering to this guideline, the research team ensured that all the diverse perspectives, themes and nuanced aspects of the research topic were explored comprehensively, and no new themes or insights emerged at this point. To ensure the validity, transparency, and reliability of the findings, this research applied specific criteria and measures such as data triangulation and maintained a comprehensive database of all data sources used in the study. The methods employed to check validity are detailed in Appendix 1. Furthermore, to increase the richness of the data, the semi-structured interviews were complemented with secondary data obtained from extensive literature review and analysis of policies and industry reports (such as the UK Sixth Carbon Budget, 2020).

### 3.3 Data analysis

Data analysis began by coding the interview transcripts on NVivo 12 Qualitative Research software following the procedures recommended by Saldaña [62]. The first step was to associate the data with first-order codes related to the study's main topic: building sector practices and underlying construction industry culture towards achieving climate targets in Manchester and Melbourne. This analysis was an iterative process involving moving back and forth between the data and literature to make sense of emerging concepts, determine the different views of the experts in the two cities and to refine the coding scheme. Next, the common themes between the two datasets were used to link related categories and cluster the initial first-order codes into more precise second-order themes. For example, statements reflecting a belief that more stringent building codes were needed were noted under *‘building codes and standards.’* This process was done iteratively until theoretical saturation was reached.

In the next stage of analysis, the data was revisited to refine the provisional second-order themes and create new themes emerging from the analysis. The team discussed and refined the provisional themes to ensure that they accurately reflected the first-order codes. After finalising the second-order themes, the underlying theoretical dimensions were investigated to understand how the themes interacted and related to each other within the larger context. The themes were also analysed for differences and similarities in practices between Manchester and Melbourne. The results were scrutinised against government and industry reports as well as relevant literature to determine how well the emergent theoretical understanding explained the research setting. As suggested by Pratt [63]; the data from the analysis is presented in a structured manner in Table 3 below to illustrate how the first-order concepts and second-order categories formed the aggregate themes discussed in the Results section.

**Table 3:** First order concepts, second order categories and aggregate themes.

Representative quotes	First order concepts	Second order categories	Aggregate themes	Description
“**Generally, in industry carbon neutrality is poorly defined” [UK- Engineer 4] … “there is sort of a massive education gap to demystify carbon jargon” [Australia – Sustainability expert 4]**	Carbon neutrality definition; carbon jargon demystification	Working definitions	Decarbonising the building sector	This theme delves into the specific practices and strategies employed within the building sector to reduce carbon emissions.
**“For the longest time, we have been focusing on reducing operational energy because it was much more significant” [UK- Engineer 1]**	Operational energy focus	Scope of assessment		
**“I would not say it is a sustainability-driven thing, it is investment costs that drive decisions” [Australia – Sustainability expert 3]**	Investment-driven decision making	Decarbonisation decisions		
**“For major projects, there is an inherent allowance or expectation that there will be money for a sustainability consultant or specialist advice” [Australia- Engineer 4]**	Sustainability practices	Sustainability budgets		
**“Low carbon materials might be more expensive today because of demand and supply forces” [Australia – Sustainability expert 5]**	Low carbon materials; market forces	Construction materials		
**“Design standards have been more relaxed particularly in regard to the airtightness of buildings” [Australia – Engineer 3]**	Design standards	Building codes		
**“The Green building tools forces the designer to think through material selection and design efficiencies” [Australia – PM 4]**	Green building tools	Green building certification		
**“Our organisation is taking a proactive approach that tries to advocate for our clients to take up innovative strategies to reduce their emissions” [Australia – Engineer 6]**	Push for emission reduction	Compete-driven	Construction industry culture	This theme shifts the focus towards the cultural aspects that impact and shape the practices within the building construction industry. It explores how policies, regulations, knowledge and attitudes affect decision making processes and the implementation of carbon reduction measures.
**“The UK is predominantly driven by a design for compliance culture” [UK – Sustainability expert 1]**	Design for compliance culture	Compliance-driven		
**“The developer aims to secure anchor tenants such as government or large organisations who are only going to occupy a building that meets certain sustainability requirements” [Australia – Builder 2]**	Anchor tenants and sustainability	Market-driven		
**“A shift in the construction industry culture is necessary for transformational change” [UK- Policy expert 2]**	Cultural shift	Performance-based		

## 4 Results

This section synthesises the results of the semi-structured interviews, supplemented with secondary data sources. The research findings are categorised into two primary themes: i) decarbonising the building sector and; ii) construction industry culture. These dimensions offer insights into the prevailing practices in the building sector of the two cities, with appropriate references to their respective countries when needed. To enhance comprehension, selected quotes are incorporated within the text to illustrate the findings.

### 4.1 Decarbonising the building sector

#### 4.1.1 Definition of terms

This study noted a lack of clarity in *“carbon neutrality”* and *“net zero”* definitions in both Manchester and Melbourne. Among the participants, 24 % (6 from Manchester and 9 from Melbourne) referred to achieving reduced operational energy through improved thermal performance and offsetting the remaining emissions primarily through power purchase agreements. A further 55 % of the participants from Melbourne used the terms *“carbon-neutrality”* and *“sustainability”* interchangeably. This aligns with the perspectives of several authors (e.g. Refs. [25,64]), who highlight the nuanced interpretations and emphasise the challenges posed by the absence of a shared understanding of these terms. Such ambiguity in interpretation has been seen to make it challenging for stakeholders, policymakers, and project organisations to effectively coordinate their efforts towards achieving carbon reduction targets. The presence of only a limited number of net-zero carbon buildings in both Manchester and Melbourne could be indicative of this challenge. The sustainability experts in both cities noted that, while there is a shared ambition to deliver net zero carbon buildings, only a small percentage of the building stock is designed to meet this performance criteria. For this reason, organisations such as the Manchester Climate Change Agency have recommended a ‘Manchester Standard’ for net zero carbon new buildings to provide a roadmap for achieving this.

#### 4.1.2 Scope of assessment

The participants from both cities pointed that, although there are currently no regulations mandating embodied carbon assessment, there is a growing recognition of its contribution to the whole building life cycle emissions. This study however noted that majority of the structural engineers (78 % from Manchester and 89 % from Melbourne) were focused on embodied carbon assessment in isolation. Studies (e.g. Ref. [65]), emphasise that for a comprehensive understanding of a building's energy and carbon emissions impact, it is imperative to not only assess operational and embodied emissions independently but also to recognise the interplay between the two. By doing so, stakeholders can optimise their relative and combined impacts to avoid the unintended consequences that may arise from assessing each in isolation. Notably, when Life Cycle Assessments (LCA) are conducted on major projects in both cities, the focus was found to be on the materials rather than a whole building approach. This was attributed to the unavailability of data, a lack of expertise, and the tedious nature of the process. Policy expert 4 from Melbourne pointed out that, while databases such as EPiC have made material assessments easier, designers, architects, structural engineers and project managers rarely possess expertise in LCA. This highlights the pressing need to upskill the current workforce to bridge this knowledge gap.

Additionally, the end-of-life treatment of buildings is currently not considered in either Australia or the UK. Although there are initiatives that promote the reuse and recycling of materials, such as the Construction and Demolition Waste Guide by the Australian Department of Sustainability, Environment, Water, Population and Communities, they are currently voluntary and not yet integrated into building regulations or certification systems. One of the building services engineers noted that *“it is rare for buildings to be designed with the end in mind … With the buildings we do now, they are designed to last 100 years. Nothing is designed to last that long*” (Australia-Building Services Engineer 3). Due to technological advancements over the building's lifecycle, the nature of these processes is highly uncertain. Therefore, researchers such as Sandin et al. [66] recognise this time-dependent uncertainty as the underlying reason why end-of-life scenarios are often ignored in LCAs. However, ignoring the end-of-life treatment of a building means that the carbon emissions associated with disposing of the building materials are not included in the calculation of the building's carbon footprint, resulting in an underestimation of the building's true carbon footprint. Moreover, this can result in missed opportunities for sustainable building practices such as recycling and reusing building materials at the end of life. Therefore, there is a need for building regulations and certification schemes to integrate sustainable practices for the end-of-life treatment of buildings to achieve truly sustainable and environmentally responsible building practices.

#### 4.1.3 Decision making on decarbonisation strategies

The findings revealed that 67 % (18 from Manchester and 24 from Melbourne) of the participants identified the initial upfront cost as the primary determinant influencing the selection of decarbonisation options in building projects. Consequently, building owners and developers tend to prioritise upfront costs over the building's overall environmental performance. This emphasis on cost often overlooks innovative decarbonisation alternatives that could otherwise enhance the building's environmental sustainability. In Manchester and the broader UK context, the participants observed that decarbonisation alternatives are primarily adopted by larger clients who are willing to pay a premium to mitigate reputational risks. Within these organisations, there is an evolving perspective on the concept of value for money. This redefinition is driven by their net zero commitments and encompasses several factors, including evaluating the climate risk exposure in buildings and the possibility of long-term reputational damage.

In Melbourne, 30 % of the participants highlighted that it is common for decarbonisation initiatives to be the first to be *‘value engineered’* out when the project encounters budgetary pressures. This cost-centric approach may be attributed to traditional design and construct procurement methods where contractors/builders deliver the building and are not involved in its use phase. While decarbonisation initiatives have the potential to yield long-term cost savings through reduced energy use and lower operating costs, the developers noted that the upfront costs of implementing these initiatives are often substantial, making it challenging to justify the additional costs during the construction phase without appropriate incentives. To address this, there is increasing evidence in literature supporting the adoption of an integrated design process to develop cost-effective strategies [67]. This approach involves a holistic cost assessment of building components and systems, rather than focusing narrowly on individual line items.

### 4.2 Evaluation of decarbonisation decisions

A recurring theme that emerged in Melbourne was that, although low carbon materials were required to be selected at the initial planning and design phases to meet the project's sustainability targets, these recommendations often lacked specific quantifiable metrics and reference points for assessing performance, clear definitions of what qualified as low-carbon materials and information on how compliance would be measured throughout the project life cycle. 21 % of the participants from Melbourne observed that this deficiency in specific metrics and guidelines frequently resulted in poor coordination and communication within the teams. This not only posed challenges to team dynamics but also raised concerns about the overall effectiveness of sustainability initiatives in the building projects.

In Manchester, the empirical data revealed a disparity in the assessment of the impact of decarbonisation decisions when multiple stakeholders are engaged. For instance, one of the project management professionals noted that a mega project that they were involved in had a sustainability plan that provided an overall framework to support the principles of sustainability in the design, construction, and operation phases of the project. While this plan tied together the overarching goals and strategies, it was deficient in providing specific, step-by-step guidance for implementing and assessing performance. From a project management perspective, the absence of a clear process map results in team members holding divergent and conflicting interpretations of the “right” approach, leading to inefficiencies, as highlighted by the Royal Institute of British Architects (RIBA)[68]. Consequently, the participant noted that, despite the project's ambitious sustainability targets, it struggled to attain these targets due to the absence of a structured process map to navigate the decision-making process between numerous stakeholders.

Notably, the participants highlighted that evaluating the impact of decarbonisation strategies along the project lifecycle is still immature in both cities. *“A lot of work still needs to be done to monitor and evaluate the impact and progress of decarbonisation decisions over time and make the necessary adjustments”* [Australia - Sustainability Expert 7] … *“It is very rare that we re-evaluate the impact of decisions after the project is completed, unless when monitoring the building's energy performance*” [UK- Engineer 3]. This is perhaps because there are often insufficient resources allocated to monitor the building's performance after it is handed over. For example, the participants noted that for university buildings, performance is monitored at a campus or portfolio level, with in-depth assessments of individual buildings being undertaken only when there is a specific need to do so. This finding aligns with previous research conducted by Durosaiye et al.[69] which highlights that building professionals have not yet fully appreciated the importance of revisiting constructed buildings to assess their ongoing suitability and performance. To address this, the London Plan Policy SI2 (2021) requires major developments within its jurisdiction to continuously track and provide reports on their energy performance for a minimum duration of five years. Additionally, BREEAM Man 05 incentivises aftercare support, encompassing commissioning activities and post occupancy evaluation during the first year of the building's operation to ensure that it operates according to the design intent and in response to the occupant's needs. Both Manchester and Melbourne may benefit from considering similar policies and initiatives to address performance gap issues and enhance overall building performance.

#### 4.2.1 Project sustainability budgets

In major building construction projects, sustainability initiatives and specialist advice are typically included in the project scope alongside a sustainability budget provision. However, this study revealed divergent interpretations of this concept among participants from the two cities. For instance, Sustainability Expert 7 from Melbourne reported that the high-profile projects that they had been involved in often allocated financial resources specifically for sustainability-related activities within the project. This included resources for energy-efficient technologies, water, and waste management. In contrast, Sustainability Expert 2 from Manchester described the term *“sustainability budget”* in a different light, signifying an anticipated reduction in the project's environmental impact. While financial resources were earmarked for these strategies, the primary metric of measurement focused on carbon reductions that were aligned with the organisation's Environmental, Social and Governance (ESG) and net zero goals. With these targets gaining prominence, 27 % of the participants working in multi-national companies in the UK indicated a shift in emphasis from viewing the project's budget solely in financial terms to aligning this with the organisation's emission targets.

Although these multinationals and other progressive businesses are publicly announcing their commitment to achieving net zero goals, there remains a significant number of small to medium enterprises that are still lagging, leading to inertia in the system. At the same time, there are significant concerns within the academic community that the targets put forth are vague aspirations lacking concrete implementation strategies [70]. To put this into perspective, The Science Based Targets initiative (SBTi) [71] reports that only 220 companies in the UK have net-zero commitments and have set their near term or long-term targets. However, data from the Office for National Statistics [72] revealed that 38 % of businesses were actively implementing measures to lower their greenhouse gas emissions, with an additional 24 % expressing their intention to act within the subsequent 12 months. This situation is mirrored at project level in Australia. For instance, Structural Engineer 1 highlighted that, despite proposing innovative methods to reduce a building project's carbon impact in the business case, these measures may not always be implemented during construction viz:

*“To attract investors in the bidding phase of projects, we try to have some fluffy words around what we will do in terms of reducing the carbon impact of the project. And sometimes, it is just a tick box exercise to say, yes this is what we will do”* [Australia - Structural Engineer 1].

It is important to note that, while emission reductions are motivated by ESG targets, there are weaker links to the wider national carbon budgets, indicating a lack of policy integration between the building sector's practices at the project level and government policy. One of the engineers noted that *“it is only seven years to 2030. This does not cover a full lifecycle. The challenge is that we have not designed any buildings according to the carbon budget. In fact, we do not even know what the implication of this would be”* (Australia – Engineer 5). This lack of understanding and awareness around what a net-zero future means and looks like was further reinforced by sustainability experts from the UK viz: *“we have not designed or delivered a single building as* per *the carbon budget”* (UK- Sustainability expert 4). The lack of clarity in defining carbon budgets for buildings contributes to the limited understanding of the practical implications of delivering carbon-neutral developments [73]. Consequently, setting targets and comparing the carbon impact of projects against benchmarks becomes a challenging task. To address this issue, there is a need to translate and cascade the national carbon budget to the sectoral and project levels and collect relevant data for analysis on a whole building scale. Further, sustainability experts from the UK noted that addressing the building's carbon budget at the project level rather than the material level will provide holistic carbon reduction goals.

#### 4.2.2 Construction materials

Manchester is an industrial city facing challenges related to the carbon intensity of its existing building stock. Engineer 5 and Contractor 2 from Manchester highlighted that the focus has mainly been on upgrading the existing structures through retrofitting and installing heat pumps to achieve net-zero operational carbon performance. In contrast, ClimateWorks Australia [74] estimates that 51 % of the buildings expected to stand in 2050 in Australia will have been built after the year 2019. There is therefore a significant emphasis on decarbonising new building constructions in Melbourne. However, 70 % of the participants noted that the inadequate manufacturing capabilities for low-carbon materials in Australia is a major challenge. For instance, Engineer 2 mentioned that large building companies often rely on Chinese suppliers to provide façade components in bulk quantities, particularly for materials such as glass and aluminium. As a result, low-carbon materials may be foregone in some building projects due to long lead times that cannot be accommodated by tight project deadlines. The participants, therefore, agreed that the government should take measures to re-energise the manufacturing industry to facilitate this low-carbon transition.

Similarly, noting that low-carbon materials are generally expensive, sustainability experts in the UK advocate for stricter regulations to mandate a certain level of performance. This is because without such regulations, it can be challenging to convince investors and clients to pay the additional cost viz:

*“If getting 5% better performance in the building costs an extra 50 million pounds, you have got to convince the people paying that money that it is worth it. And, without a mandate, it is pretty hard”* [UK - Engineer 3].

This study notes that adopting low-carbon materials initially involves higher costs; however, as the demand for these materials increases, it will result in economies of scale. Consequently, there will be a downward shift in the cost curve, facilitated by the allocation of additional factory floor space towards the production of low-carbon materials. This increased demand is exemplified by the progress made in adopting solar photovoltaic (PV) technology for on-site renewable energy. Notably, Engineer 3 from Australia observed that a decade and a half ago, installing a rooftop solar PV system required a business case to demonstrate its economic viability to stakeholders. At the time, the payback period in Australia was a decade. However, with the increased demand for on-site renewable energy options, this has now decreased to four years. As a result, solar power has evolved into a standard design requirement in most cases, eliminating the need for a business case. This surge in PV adoption aligns with data from the Australian PV Institute [75]; which reports over 3.52 million PV installations in Australia with a combined capacity exceeding 32.1 GW in June 2023, compared to 12,829 PV installations with a combined capacity of 23, 329 kW in June 2008. This trend can be attributed to various factors, including innovation, competition, public policy, and growing concerns about climate change [76].

Besides this, the participants highlighted other challenges related to the availability of Environmental Product Declarations (EPDs). The Australian building sector relies heavily on the electricity grid, which was responsible for 50.1 % of the total carbon emissions in Victoria in 2020 [77]. Given that a significant portion of the energy demand is primarily met by coal-generated power [43], the participants observed that Australian-based manufacturers *(supply side)* have been hesitant to disclose their EPDs due to concerns that their products may have a higher embodied carbon content compared to their international counterparts. On the *demand side*, the integration of EPDs into the initial phases of competitive tender processes without negatively impacting competitiveness or causing price hikes was highlighted as another challenge. Participants noted that when contractors or suppliers are required to use products with EPDs during the tender process, it imposes strict limitations that ultimately deter competition and inflate market prices. Therefore, collaborative effort between industry players, regulatory bodies and environmental organisations is important to develop industry-wide EPDs that provide a benchmark for assessment.

#### 4.2.3 Building codes and standards

Engineers in Melbourne and Manchester expressed concerns regarding the challenges of designing energy-efficient buildings considering climate change, noting that the current building codes and standards reward the status quo. In Melbourne, the engineers noted that Australia lags behind the UK in its minimum design standards for residential buildings, particularly in terms of air sealing, energy, and thermal comfort. This has led to more relaxed design practices for energy efficiency, resulting in increased energy consumption for heating and cooling. In some cases, designers prioritise aesthetics over energy efficiency viz:

*“We use film glass and for the four months that it is cold, we just turn the heater up … which is unsustainable because we are burning more gas. I think sometimes we are a bit short-sighted that we focus on aesthetics and not worry too much about heat loss because you will find other ways of dealing with it”* [Australia – Engineer 3].

Further, a sustainable building advisor who closely collaborates with builders in Melbourne observed that, although building energy costs have been on the rise, insulation is still overlooked in residential buildings due to the belief that installing the required heating and cooling systems would compensate for the lack of insulation. This perspective stems from construction cost considerations, as many builders are unwilling to invest extra costs since they do not benefit from the building's operational savings. 75 % of policy experts in Melbourne also recognised this prevailing resistance among small residential builders towards building insulation, primarily due to concerns regarding associated risks such as fire incidents and fatalities. This underscores the need for measures to de-risk insulation as well as establish minimum standards for insulating rental homes.

On the other hand, participants from Manchester expressed concern that current building practices do not account for the uncertainties associated with potential future climate changes. Manchester's colder climate has historically emphasised insulation and heating-related energy efficiency measures. Consequently, the conventional approach has prioritised achieving the highest insulation performance for building façades. However, without proper shading to prevent unwanted solar heat gains, buildings are exposed to the risk of overheating during the hotter summers. Due to these challenges, 87 % of the interviewed participants agreed that there is a need for holistic design standards that form part of the standard procurement process and serve as a benchmark for measuring performance and consistency.

#### 4.2.4 Green building certification

**Fig. 1:**
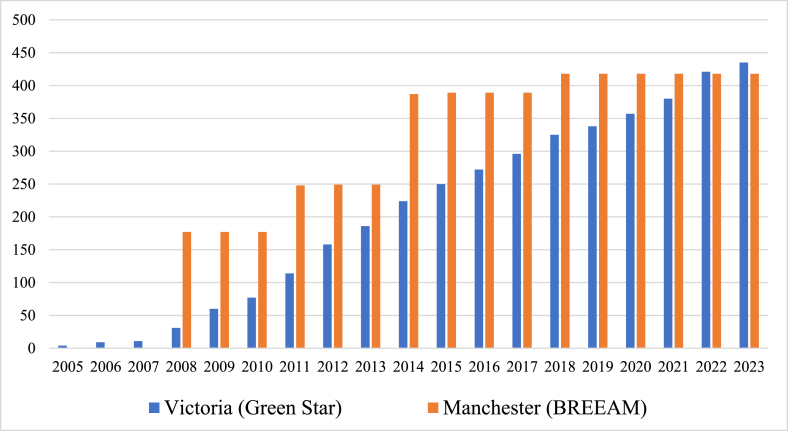
Cumulative number of GBR rated buildings in Manchester and Melbourne (Source: Data extracted from [11,41]).

The participants noted that carbon reduction in the building sector is primarily driven by green building certification. Notably, Building Research Establishment Environmental Assessment Method (BREEAM) in the UK and Green Star (Australia) have witnessed substantial adoption, as depicted in Fig. 1. In Manchester, 36 % of these certifications are commercial buildings, 23 % office buildings, 19 % Higher Education buildings, 9 % in residential buildings and the remaining 13 % are community centers and other types of buildings. In Victoria (Melbourne), the distribution is 13 % commercial buildings, 45 % office buildings, 13 % in Higher Education buildings, 6 % in multi-residential buildings, and the remaining 23 % accounts for community centers and other building types as of 2023.

An alternative commercial building rating system, National Australian Built Environment Rating System (NABERS) has acted as a driving force for market transformation in Australia, motivating *supply-side actors* to incorporate operational energy performance into the design process through the base building rating. This has subsequently impacted investment decisions, as tenants *(demand side actors*) are hesitant to occupy new buildings unless they can be assured of the operational energy building rating. Consequently, investors and developers have been compelled to provide guarantees regarding the expected energy usage of newly constructed office buildings. The participants in the study observed that in Australia, office buildings consistently achieve measured building performance that aligns with design predictions. In fact, developers can register for a NABERS commitment agreement, targeting a specific NABERS rating and carbon intensity per square meter. This agreement can then be used to advertise to potential tenants before the building is operational and the tenants are required to fulfil certain obligations as part of the commitment agreement.

On the other hand, the participants from Manchester noted that the majority of the projects they were involved in were primarily focused on achieving BREEAM credits to meet their organisational sustainability targets. However, studies have challenged the notion that green building certifications inherently lead to superior energy efficiency or reduced carbon emissions. For instance, Hu [78] conducted a study on LEED and non-LEED office buildings in Washington DC and found that LEED office buildings collectively used 17 % more source energy and 13 % more site energy than non-LEED buildings. Furthermore, Matisoff's [79] research suggests that some building owners pursue LEED certification primarily for the prestige of the certification rather than closely adhering to specific environmental or sustainability criteria.

### 4.3 The construction industry culture

#### 4.3.1 Compete-driven culture

**Fig. 2:**
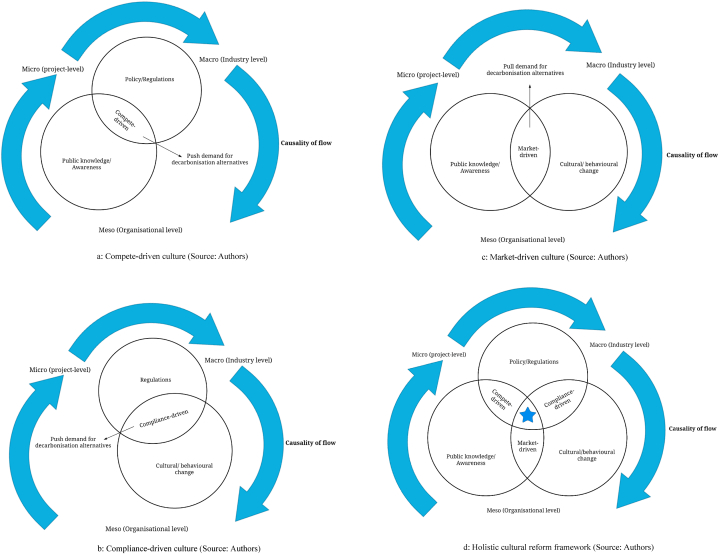
a: Compete-driven culture (Source: Authors). b: Compliance-driven culture (Source: Authors). c: Market-driven culture (Source: Authors). d: Holistic cultural reform framework (Source: Authors) *The star in (d) represents the performance-based culture, which is the gold standard for the industry achieved through the combination of push and pull interventions.

This study found that, as voluntary policy instruments and certification schemes come into place, the level of awareness of climate issues and targets increases, and generally has the potential to facilitate positive change. At this point, forward-thinking investors and clients who are willing to pay the cost premium to achieve ambitious goals drive this change by pushing the demand for low-carbon solutions, creating a *‘compete-driven’ culture* as depicted in Fig. 2a. This culture was found to be particularly evident in Australia's residential building sector, where low-carbon solutions are predominantly utilised in high-value projects to attract premium tenants. Conversely, smaller builders prioritise profits rather than actively seeking a competitive advantage. Since low-carbon solutions are still costly at this point, it is critical to establish long-term regulatory frameworks that enforce stringent standards to provide both certainty and a level-playing field to enable the actors in the supply chain to innovate and hence reduce costs [80]. Further, a cohesive set of regulations and financial considerations such as rebates and incentives should be put in place to allow for a stable carbon-neutral economy to emerge [81].

Notably, the research participants highlighted that *“although policies are slowly coming in, there is still a massive education gap to demystify carbon jargon”* [Australia - Sustainability Expert 5]. To address this issue, public knowledge and awareness can be influenced by: i) providing industry expertise through updated curricula in learning institutions and training programs for upskilling the current workforce; ii) disseminating information to the public (end-users and clients) through various avenues, such as social marketing and; iii) greater collaboration between research institutions and industry actors [82].

#### 4.3.2 Compliance-driven culture

Compliance has traditionally been defined as conformity to legislation and regulations [83]. Scholarly works further suggest that this can be achieved when stakeholder expectations influence behaviour [84]. Therefore, this study argues that when the behaviours of building sector professionals are modified in a way that aligns with legal obligations to avoid stakeholder scrutiny, then this is predominantly a '*compliance-driven'* *culture*. While policy mechanisms can be in the form of regulatory measures, voluntary instruments and economic incentives [85], the findings revealed that sustainability experts in both cities favour stricter regulations whereas developers and builders prefer subsidies and incentives over penalties. Notably, one participant suggested that a combination of regulatory measures and market-based incentives may be more effective in promoting decarbonisation alternatives, stating that *“in a developed economy, regulators should try to find a bit more carrots and fewer sticks … and if there is a market way of generating that, then it is more likely to be successful than regulation alone”* (Australia - Sustainability expert 5). This can be exemplified through the enactment of Local Law 97 by the New York City Council [86]. The legislation introduced emissions intensity limits for various building typologies, with the aim of achieving 80% reduction in GHG emissions across all New York City's building stock by the year 2050. The initial emission limits are set to be enforced from 2024 to 2029, followed by even stricter limits from 2030 to 2034, accompanied by increased financial penalties. This gradual implementation timeline *(carrots)* offers property owners an opportunity to assess their building portfolios and develop strategies to comply with these requirements [87]. Under Local Law 97, a penalty of $US268 (approximately $A358) per metric tonne of CO_2_e exceeding the specified limit was instituted *(sticks)*. This penalty amount was determined based on the “social cost" of carbon.

It is generally postulated that direct regulation by the government has the potential to facilitate the transition in the building sector. The European Union's Energy Performance of Buildings Directive (EPBD) is a prime example of a policy objective that aimed to achieve nearly zero-energy housing (ZEH) by 2020 through best practice [88]. While deliberate intervention by the government has the potential to act as a catalyst for triggering disruptive innovation in building design, its effectiveness is limited, as evidenced by Koeppel & Ürge-Vorsatz [89]. This study highlights that the implementation of policies aimed at driving behavioural change towards climate targets, as observed in the UK, can give rise to a *“compliance-driven”* culture within the building construction industry (refer to Fig. 2b). This culture tends to foster prescriptive-based design practices focused on meeting minimum requirements rather than seeking innovative approaches to optimise environmental performance. For instance, building design regulations that establish energy performance standards for buildings in the UK such as ‘Part L’ emphasise reduced energy demand during the design phase. However, this *“design for compliance”* culture often leads to buildings that meet minimum compliance requirements in the design phase without sufficient consideration for the operational phase, resulting in performance gap issues [90]. Furthermore, regulatory compliance does not equal quality compliance nor best practice [91].

#### 4.3.3 Market-driven culture

The study found that multinational consultancies in the building construction sector in both cities are leading the way by *“pushing”* for the adoption of carbon-reduction strategies. However, participants in the study highlighted that a comprehensive approach would require a *“pull system,”* where clients and end-users actively demand such strategies, leading to increased adoption. For instance, one builder noted that,

*“Often for large office building developments, the developer aims to secure anchor tenants such as government or large organisations. These organisations are only going to occupy a building that meets certain sustainability requirements. In this case, even when there are cost pressures, you still must meet the requirements of the anchor tenant. Otherwise, the project is not financially viable at all.”* [Australia – Builder 2].

For this reason, performance benchmarking policies, such as the NABERS in Australia was particularly seen as effective in promoting the benefits of energy efficiency in the commercial office sector, thus creating a *“market pull*.” As public awareness grows, there is an increased demand for zero-carbon building developments from clients and occupants, leading to a transition in the building sector toward a *“market-driven” culture* (refer to Fig. 2c). This pressure from investors, occupants, clients, and internal stakeholders has thus accelerated the pace of change towards carbon reduction in the commercial office building sector resulting in high performance, premium and A-grade office buildings that are delivered to meet sustainability targets.

Once this precedent is established, design standards that meet high-performance criteria became the norm and a contractual requirement. Achieving this culture necessitates behavioural changes that involve adjusting daily choices and practices. To facilitate rapid transformation in the building sector practices, applying behavioural insights, such as nudges as explained by Bukoye et al.[53]; can be effective.

## 5 Discussion

Despite the climate emergency declarations in both Manchester and Melbourne in 2019, along with national commitments to achieve net-zero emissions by 2050, the building sector transformation has been relatively slow in the two cities. The empirical analysis revealed that the climate emergency understanding is embedded in plans and policy documents in both cities (such as Manchester City Council Climate Change Action Plan and Victoria's Climate Change Adaptation Plan), but little explicit attention has been given to the processes of prioritisation and mobilisation towards the Paris 2050 challenge at the project level. Too et al. [25] assert that the building sector must adopt a *performance-based culture* that considers the environmental impact of the building throughout the entire project lifecycle if it is to make substantial strides towards achieving climate targets.

Although government regulatory and incentive programs have been instrumental in initiating positive changes such as retrofitting older buildings in the UK, it is essential to supplement these efforts with initiatives that raise public awareness to increase the adoption of sustainable practices [92]. There is therefore a growing recognition for a more holistic approach that considers the underlying social and behavioural factors since they significantly influence the effectiveness of these policies and the adoption of technologies [93]. This study argues that policy and regulatory frameworks, knowledge enhancement and cultural change are the key building blocks for cultivating this transformational change. Drawing from extensive literature and empirical findings, the following section elucidates how these three drivers collectively foster a *performance-based* culture.

### 5.1 A performance-based culture

As elaborated upon in earlier sections, pushing demand for low-carbon solutions through regulations and design standards can create a level playing field by setting minimum standards for sustainable practices, ensuring that all organisations meet a basic level of environmental performance *(compete-driven and compliance-driven cultures)*. However, it is sometimes perceived as burdensome and may not be sufficient to motivate organisations to go beyond the minimum requirements [94]. In contrast, pull demand driven by market forces encourages organisations to adopt sustainable practices voluntarily [95]. This can lead to innovation and more ambitious sustainability targets, as organisations seek to differentiate themselves and gain a competitive advantage *(market-driven culture)*. However, this study notes that this results in a fragmented approach to sustainability, with some organisations leading the way while others that are not willing to pay the premium price lag behind. Therefore, the synergetic application of these three drivers is necessary to influence the building construction culture towards a *“performance-based” culture*.

Traditionally, building design solutions were driven by prescriptive terms that involved specifying the properties of the solution rather than the expected performance of the design solution [96]. This was primarily driven by building codes and regulations which offered prescriptive specifications based on minimum specific requirements such as load resistance, ventilation rates, and wastewater specifications [97]. However, authors (e.g. Ref. [91]), argue that these prescriptive approaches may not reflect best practices and can hinder progress toward climate targets. The transition from *prescriptive models* to *performance-oriented frameworks* therefore emerged in response to the realisation that prescriptive approaches stifle innovation, hinder cost-effectiveness, and impede global knowledge exchange [98]. Performance-based building regulations, also known as function-based or objective-based building standards, codes, or regulations, were first introduced in the early 1980s [99]. Since then, these regulations have gained traction globally. For instance, Victoria's Environmentally Sustainable Development (ESD) of buildings and subdivisions requirements promote a performance-based approach by obliging developers to meet specific environmental performance targets related to energy, water, and waste reduction [100].

Drawing on the empirical findings and best practice guidance, this study argues that a set of interconnected *“push and pull”* interventions that meet the *“carrot and stick”* approach should be implemented to bring about a *“performance-based” culture* (refer to Fig. 2d). Pull interventions focus on incentives such as financial rewards, public recognition and knowledge sharing initiatives while push interventions focus on pressures as a result of stricter regulations, contractual penalties and regular performance reviews. For complete transformation of the building sector, this cultural shift should occur along the entire value chain that is, macro (industry level), meso (organisational level) and micro (project level). This causality flows in both directions between the macro and micro, making it possible to intervene at different levels to create change. Taking this socio-technical perspective towards policy development in the building sector will enable the incorporation of cultural dimensions that the conventional economically oriented approach to policy formulation cannot adequately tackle [101].

## 6 Conclusion

Cross country comparisons play a crucial role in highlighting both similarities and disparities that countries must consider when formulating their net zero pathways [17]. While studies (e.g. Refs. [102,103]), conduct cross-country comparisons, their focus has been limited to energy consumption patterns. There is therefore a gap in research directly comparing building sector practices in response to climate emergency declarations. This study bridges this gap and contributes to the current body of knowledge on decarbonisation and sustainability efforts by examining primary data obtained from in-depth semi-structured interviews with building professionals and policy experts from two major cities. The study not only uncovers the multifaceted challenges faced in decarbonising the building sector but also sheds light on effective strategies toward fostering a *performance-based culture*. Through this comprehensive study, this research enriches the academic discourse on the building sector response to climate change, offering insights that can guide policymakers, researchers and building sector practitioners towards achieving net-zero carbon buildings. It also emphasises the significant influence of the construction industry's culture on achieving climate targets.

The research findings suggest that a narrow focus on regulation often results in a *compliance-driven culture* that is driven by the need the need to meet minimum requirements. Shifting societal behaviours and industry culture towards a low-carbon future requires a holistic approach that integrates policy instruments *(regulations and incentives)* with knowledge exchange platforms. For instance, carbon taxes, alongside well-designed incentives, can nudge behavioural changes by internalising environmental costs and promoting sustainable choices. When complemented by robust knowledge exchange between stakeholders, this approach fosters long-term cultural transformation in the sector, enabling the transition to low-carbon design and development practices. Additionally, a reflexive governance system is needed at the project level to conduct periodic reviews and assessments to ensure the building's carbon targets remain on track, thereby avoiding ‘locking in’ practices that could hinder the achievement of the goal.

Based on the empirical data, this paper proposes a holistic cultural reform framework that illustrates how policies and regulations, public knowledge and behavioural change can collectively foster a *performance-based culture* in the building sector. The framework goes beyond the prevailing technical and policy-focused discourse in this research area, contributing to the advancement of the carbon transition discourse by providing a clear pathway for moving away from traditional business-*as*-usual practices towards strategic cultural changes in the built environment. Future research could explore the effectiveness of the proposed cultural reform framework in different contexts and jurisdictions.

## CRediT authorship contribution statement

**Judy Too:** Writing – review & editing, Writing – original draft, Project administration, Methodology, Investigation, Formal analysis, Data curation, Conceptualization. **Obuks A. Ejohwomu:** Writing – review & editing, Supervision, Conceptualization. **Felix K.P. Hui:** Writing – review & editing, Supervision, Project administration, Conceptualization. **Nilupa Herath:** Writing – review & editing, Supervision. **Colin Duffield:** Writing – review & editing, Supervision.

## Declaration of competing interest

The authors declare that they have no known competing financial interests or personal relationships that could have appeared to influence the work reported in this paper.

## Figures and Tables

**Table:** 

Test	Tactic	Implementation
Content validity	Using experts to assess the content of the research instruments	To establish content validity, the interview questions were carefully designed and aligned with the research objectives. The questions covered key dimensions related to carbon reduction practices in the building sector of Manchester and Melbourne. Prior to the main interviews, a panel of experts reviewed the interview questions to ensure their relevance and appropriateness.
Construct validity	Using multiple evidence sources to establish a chain of evidence	The construct validity of the interview responses was evaluated through a qualitative data analysis approach. Thematic analysis was conducted on the interview data to identify patterns and themes related to carbon reduction practices in both cities. The identified themes were then compared with the theoretical dimensions of the research, confirming the alignment between the data and the intended constructs. Interviews were supplemented with evidence obtained from industry and government reports.
Internal validity	Pattern building	Findings from the interview transcripts and secondary data were analysed to identify patterns in building sector practices in Manchester and Melbourne. Existing literature were used to explain emerging findings.
External validity	Replication logic	Building experts involved in different projects were selected to ensure that the findings were generalisable. Data was drawn from two representative cities to provide building sector practices and recommendations that are generalisable.
Reliability	Using an interview protocol Project database	An interview protocol that contained interview questions to guide the discussion was developed. A database was created to store data from both primary and secondary sources.

## Footnotes

1 Ethics clearance was obtained from the Human Research Ethics Committees at both the University of Manchester and the University of Melbourne. Approval for the research was granted by the University of Manchester's Research Ethics Committee, with reference number 2021-10515-19907, and by the Human Ethics Team at the University of Melbourne, under Project ID number 22419.
